# Presentation of an Approach on Determination of the Natural Frequency of Human Lumbar Spine Using Dynamic Finite Element Analysis

**DOI:** 10.1155/2019/5473891

**Published:** 2019-01-02

**Authors:** Fan Ruoxun, Liu Jie, Liu Jun, Wang Weijun

**Affiliations:** ^1^Department of Automotive Engineering, Jilin Institute of Chemical Technology, Jilin 132022, China; ^2^No. 2 Hospital of Jilin University, Jilin University, Changchun 130025, China

## Abstract

Occurring resonance may negatively affect the health of the human lumbar spine. Hence, vibration generated in working and living environments should be optimized to avoid resonance when identifying the natural frequency of the human lumbar spine. The range of the natural frequency of the human lumbar spine has been investigated, but its specific numerical value has not been determined yet. This study aimed at presenting an approach based on resonance for predicting the specific numerical value of the natural frequency of the human lumbar spine. The changes in the numerical fluctuation amplitudes and the cycles of lumbar mechanical parameters during resonance are greater than those during nonresonant vibration. Given that the range of the natural frequency has been identified, vibrations at different excitation frequencies within this range can be applied in a human lumbar finite element model for dynamic finite element analysis. When the excitation frequency is close to the natural frequency, resonance occurs, causing great changes in the numerical fluctuation amplitudes and the cycles of lumbar mechanical parameters. Therefore, the natural frequency of the lumbar finite element model could be back-calculated. Results showed that the natural frequency of the established model was 3.5 Hz. Meanwhile, the closer the excitation frequency was to the natural frequency, the greater the changes in the numerical fluctuation amplitudes and cycles in the parameters would be. This study presented an approach for predicting the specific numerical value of the natural frequency of the human lumbar spine. Identifying the natural frequency assists in finding preventive measures for lumbar injury caused by vibration and in designing the vibration source in working and living environments to avoid approximating to the natural frequency of the human lumbar spine.

## 1. Introduction

Lumbar degeneration is a spine degenerative disorder and one of the common causes of low-back pain [1, 2]. Meanwhile, vibration is one of the important factors leading to lumbar degeneration [3]. Vibrational environment has a nonnegligible influence on human lumbar tissue, and even long-term vibration in low frequency and small amplitude may cause or accelerate lumbar degeneration [4]. Resonance occurs when the excitation frequency is close to the natural frequency of the human lumbar spine. The force and deformation in the lumbar during resonance are more serious than those in other vibrations [5, 6]. Therefore, we should reduce vibrating time to decrease the incidence of lumbar degeneration. If a vibrational environment is inevitable, the vibration source should be optimized to at least prevent resonance.

To optimize the vibration source and subsequently prevent resonance, we need to determine the specific numerical value of the natural frequency of the human lumbar spine first. Previous experiments measured that the natural frequency of the human lumbar spine was within 1–6 Hz [7, 8]. At present, the natural frequency of the human lumbar spine is mainly predicted by dynamic finite element (FE) analysis because of the restrictions in the law and human lumbar specimen. Several FE analyses have predicted and narrowed the range of the natural frequency to 2–5 Hz [9–11]. However, applying dynamic FE analysis to precisely predict natural frequency is challenging. The human lumbar spine is composed of vertebral bone, intervertebral disc, and ligament. The bones and soft tissues cannot be simulated with the same material parameter. According to vibration theory, the mechanical properties of a structure composed of multiple materials are difficult to predict by dynamic FE analysis because the elastic moduli in each material cannot be distributed in the structure [12, 13]. Therefore, the specific numerical value of the natural frequency of the human lumbar spine is difficult to precisely predict using conventional FE analysis.

To overcome the limitations in conventional FE analysis, we intended to predict the natural frequency of the human lumbar spine based on resonance, that is, the changes in the numerical fluctuation amplitudes and the cycles of lumbar mechanical parameters caused by resonant vibration are evidently greater than those caused by other nonresonant vibrations [6, 9, 14]. Once the range of the natural frequency of the human lumbar spine is known, the excitation frequency that induced resonance can be explored, and this excitation frequency should be close to the natural frequency of the human lumbar spine.

Accordingly, this study aimed at presenting an approach to predict the specific numerical value of the natural frequency of the human lumbar spine. A poroelastic FE model of human lumbar spinal segments L2–L3 was primarily established based on healthy human computed tomography (CT) images. Vibrations at the excitation frequencies of 2–5 Hz, which was within the range of the natural frequency of the human lumbar spine, were applied to the lumbar FE model to perform time-dependent dynamic FE analysis. The excitation frequency, which may induce resonance, was investigated by observing the changing processes of the lumbar mechanical parameters under different vibrations. This frequency was then utilized to predict the specific numerical value of the natural frequency of the human lumbar spine.

## 2. Materials and Methods

### 2.1. Establishment of the Lumbar FE Model

**Figure 1:**
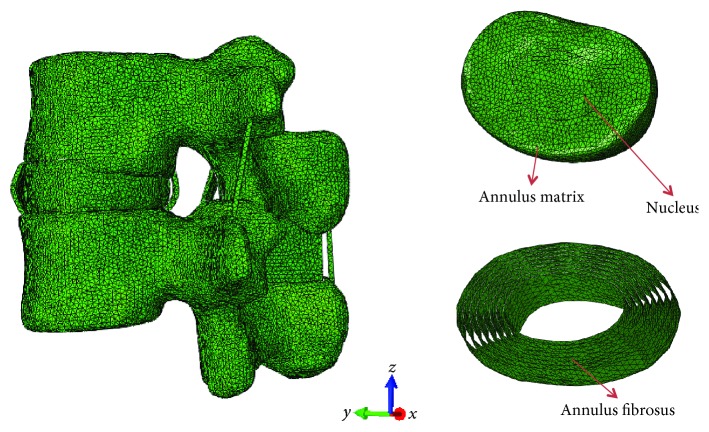
The schematic diagram of the poroelastic lumbar FE model of L2–L3 segments.

A healthy young volunteer was selected to conduct lumbar spine image scanning. Images with a resolution of 1 mm were imported into Mimics software (Materialise, Leuven, Belgium) to reestablish the 2D outside surface of the lumbar spine. Then, the 2D surface was converted into a 3D lumbar FE model using tetrahedral element with ABAQUS software (Simulia, Providence, USA). Most of the geometric appearances in the FE model were derived from the images. The soft tissues that cannot be developed during scanning were created and assembled using CATIA software (Simulia, Providence, USA) [15]. The intervertebral disc structure included annulus ground substance, nucleus pulposus, and annulus fibrosus. In particular, the annulus fibrosus was modeled as fiber-reinforced composite, which was embedded in the annulus ground substance in eight layers [16, 17]. The established lumbar FE model was shown in Figure 1.

### 2.2. Assignment of Lumbar Material Parameters in the FE Model

Porous seepage-stress coupled dynamic FE analysis was performed on the established lumbar FE model, which needs to assign biphasic materials including solid and porous parameters [15, 18]. Most of the lumbar spinal structures were assigned with the biphasic materials, except for the ligaments, facets, and annulus fibrosus, which were assigned with the solid phase [19]. The solid phase in the vertebral bone was simulated with linear elastic material; in the annulus, ground substance and nucleus pulposus were assumed as neo-Hookean hyperelastic materials [20]. The nonlinear stress-strain relationships among each ligament were fitted from a previous experiment, and the elastic moduli of the annulus fibrosus were obtained from literature [21, 22]. All the porous material parameters were selected based on literature [19, 23, 24]. The specific material parameters were shown in Table 1.

Permeability was set to be changed with void ratio and can be directly implemented into the ABAQUS code by providing the calculated permeability values for a range of allowable void ratios. The variable permeability formulation was taken from literature [18]. The permeability *k* is dependent on void ratio *e*:

(1) $$\begin{array}{c} k=k_{0}{\left[\frac{e\left(1+e_{0}\right)}{e_{0}\left(1+e\right)}\right]}^{2}\exp\left[8.5\left(\frac{1+e}{1+e_{0}}-1\right)\right], \end{array}$$

where *k*_0_ is the initial permeability and *e*_0_ is the initial void ratio.

### 2.3. Prediction on the Natural Frequency of Human Lumbar Spine

The mechanical properties in the lumbar spine are determined by the solid and porous material parameters [15, 17]. In this study, the axial effective stress and maximum radial strain in the intervertebral disc were used to express the solid mechanical properties. Pore pressure due to compression in the intervertebral disc was used to express the porous mechanical properties. The natural frequency of the human lumbar spine is within 2–5 Hz; thus, the excitation frequency was set within 2–5 Hz [9–11]. The interval was set to 0.5 Hz; therefore, the excitation frequencies of the applied vibrations were 2, 2.5, 3, 3.5, 4, 4.5, and 5 Hz. According to the dynamic FE analysis results, the changing processes of axial effective stress, the radial maximum strain, and the pore pressure in the lumbar FE model under vibrations at abovementioned excitation frequencies were observed and investigated. The excitation frequency that caused the greatest changes in the numerical fluctuation amplitudes and the cycles of lumbar mechanical parameters was subsequently identified, which can be considered as the natural frequency of the established lumbar FE model.

### 2.4. Boundary and Loading Conditions

The lower surface of the L3 segment was fixed in all directions. To apply the vibrational load, we built a reference point above the lumbar FE model and coupled with the upper surface of L2 segment. The five degrees of freedom of the reference point other than the loading direction were constrained. According to literature, for a weight of 70 kg, approximately 800 N of load is compressed on the lumbar spinal segments L2–L3 while sitting on a chair with backrest, and the vibrational amplitude range is ±10% of the static pressure [25, 26]. Therefore, sinusoidal vertical vibrations within the range of 720–880 N pressure at the excitation frequencies of 2, 2.5, 3, 3.5, 4, 4.5, and 5 Hz were applied at the reference point. The vibrational time was set to 1 hour, and a damping ratio of 0.08 was adopted [11]. The facet contacts were set to frictionless interaction, and other contacts among the vertebral bodies, intervertebral disc, and ligaments were all defined as “TIE” interaction in the ABAQUS software. Healthy disc swelling occurs because of osmotic potential and thus was simulated by a 0.2 MPa boundary pore pressure at the outer surface of the spine [17].

## 3. Results

### 3.1. Lumbar FE Model Validation

**Figure 2:**
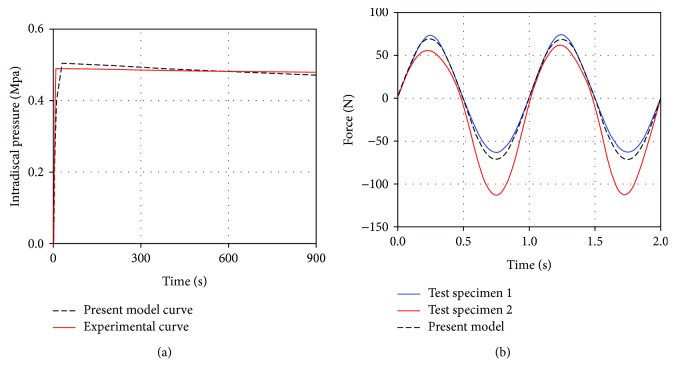
Comparison of the mechanical responses between the established lumbar FE model and the experiments. (a) Static loading condition; (b) vibrational loading condition.

First, the FE model was validated under static loading condition. The intradiscal pressure in the lumbar spine under a compressive force of 500 N for 15 min was compared with the in vitro experiment [27]. As shown in Figure 2(a), both the experimental and simulated results showed that the intradiscal pressure increased to the highest point first and then slightly decreased. Meanwhile, the maximum value of the intradiscal pressure predicted by the simulation was consistent with the experimental results.

Second, the FE model was validated under vibrational loading condition. The model was loaded at a sinusoidal anterior-posterior displacement of 0.6 mm with 1 Hz excitation frequency [28]. The predicted force-displacement curve was consistent with the curve shape of the specimens in the experiment, and the vibrational force with time was also in the range of the experimental results, as shown in Figure 2(b).

### 3.2. The Effects of Different Excitation Frequencies on the Axial Effective Stress

**Figure 3:**
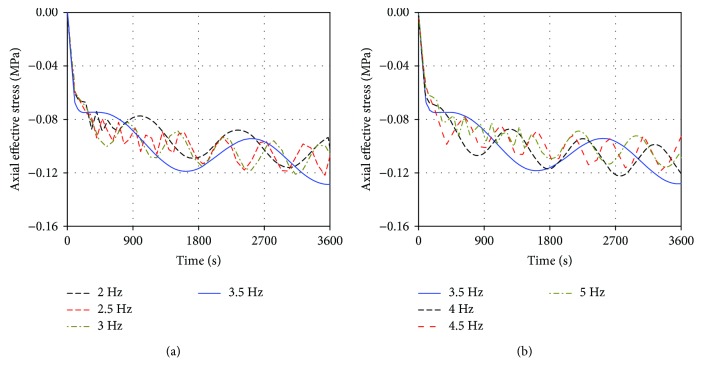
The effects of vibrations at different excitation frequencies on the average axial effective stress in the intervertebral disc.

Figure 3 illustrates the effects of different excitation frequencies on the average axial effective stress in the intervertebral disc with vibrational time (average axial effective stress was obtained by dividing the total value in the axial effective stress of all elements by the total element number in the intervertebral disc, including annulus ground substance and nucleus pulposus). Vibrations at different excitation frequencies led to great differences in the numerical fluctuation amplitude and the numerical changing cycle. When the excitation frequency increased, the numerical fluctuation amplitude of axial effective stress initially increased; when the excitation frequency increased to 3.5 Hz, the peak of stress reached the maximum; and when the excitation frequency continued to increase, the numerical fluctuation amplitude and the maximum axial effective stress decreased. The 3.5 Hz vibrational curve expressed only one complete numerical changing cycle in an hour, and all the numerical changing cycles of axial effective stress in the remaining curves were shorter than those in the 3.5 Hz vibration.

### 3.3. The Effects of Different Excitation Frequencies on the Pore Pressure

**Figure 4:**
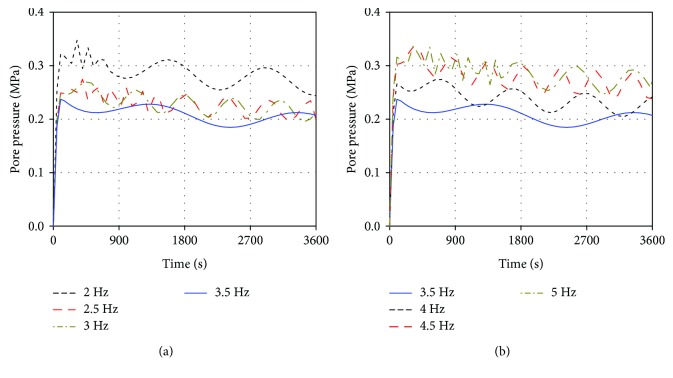
The effects of vibrations at different excitation frequencies on the average pore pressure in the intervertebral disc.

Figure 4 shows the effects of different excitation frequencies on the average pore pressure in the intervertebral disc with vibrational time (average pore pressure in the intervertebral disc was obtained by dividing the total value in the pore pressure of all elements by the total element number in the intervertebral disc, including the annulus ground substance and nucleus pulposus). The pore pressure under different vibrations could be divided into three levels: the values under 2, 4.5, and 5 Hz vibrations were at the maximum level; the values under 2.5, 3, and 4 Hz vibrations were centered; and the value caused by 3.5 Hz vibration was at the minimum level. The shapes of the pore pressure curve showed that vibration at a relatively low excitation frequency produced a long numerical changing cycle, and the shortest numerical changing cycles appeared at the 5 Hz vibrational curve. The pore pressure curve of 3.5 Hz vibration was different from other curves. Although the pore pressure at 3.5 Hz vibration changed periodically, the numerical changing cycle was long and only showed one complete cycle. Meanwhile, the difference between the maximum and minimum values of the pore pressure in this cycle was less than that in other curves, and the pore pressure continued to decrease with periodic change, thus possibly leading to an increasing gap of pore pressure value between 3.5 Hz and other excitation frequency vibrations.

### 3.4. The Effects of Different Excitation Frequencies on the Radial Strain

**Figure 5:**
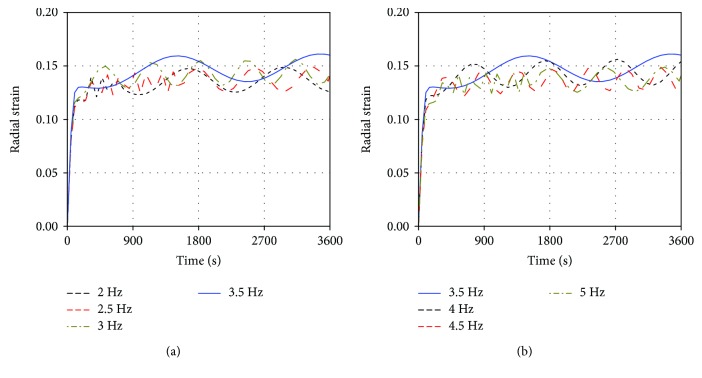
The effects of vibrations at different excitation frequencies on the maximum radial strain in the intervertebral disc.

Figure 5 shows the effects of different excitation frequencies on the maximum radial strain in the intervertebral disc with vibrational time. At the initial stage of loading, all the strain curves elevated linearly, reached a high value in a short time, and finally slowed down. When the excitation frequency increased, the numerical fluctuation amplitude and the maximum radial strain gradually increased; when the excitation frequency increased to 3.5 Hz, the peak in the strain reached the maximum; and when the excitation frequency continued to increase, the numerical fluctuation amplitude and the peak gradually decreased. The 3.5 Hz vibrational curve expressed only one complete numerical changing cycle in an hour; all the numerical changing cycles of the radial strain in the remaining curves were shorter than those in the 3.5 Hz vibration. The radial strain varied less with excitation frequency, and the differences in the value of strain generated by vibrations at different excitation frequencies were smaller than the changes in the values of the axial effective stress and pore pressure.

## 4. Discussion

The excitation frequency generated by operating transportation, such as cars, can be controlled through optimization design. However, the specific numerical value of the natural frequency of the human lumbar spine must be determined rather than merely identifying the range. Therefore, this study aimed at predicting the specific numerical value of the natural frequency of the human lumbar spine according to the phenomenon of resonance. Vibrations at the excitation frequencies of 2–5 Hz were applied to the lumbar FE model. The excitation frequency, which induced resonance, was investigated by observing the changing processes of the lumbar mechanical parameters under different vibrations. This frequency was then used to predict the specific numerical value of the natural frequency of the human lumbar spine.

The changing processes of the axial effective stress, maximum radial strain, and pore pressure in the intervertebral disc under vibrations at different excitation frequencies were observed. As shown in Figures 3–5, although the three mechanical parameters showed different values, the changing trends with the excitation frequency were similar. Thus, the axial effective stress was used as an example to discuss the vibrational change process. When the excitation frequency increased from 2 Hz to 3.5 Hz, the numerical fluctuation amplitudes in the vibrational curves increased and reached the maximum level at 3.5 Hz. When the excitation frequency increased from 3.5 Hz to 5 Hz, the numerical fluctuation amplitudes decreased. Meanwhile, the peaks of axial effective stress under vibrations at 2, 4.5, and 5 Hz were at the minimum level within 0.117–0.119 MPa. Under vibrations at 2.5, 3, and 4 Hz, the peaks were at the moderate level within 0.121–0.123 MPa. Under vibrations at 3.5 Hz, the peak was at the maximum level at 0.129 MPa. Furthermore, the longest numerical changing cycle occurred at 3.5 Hz, and the cycles at 3 and 4 Hz were longer than those at 2.5, 4.5, and 5 Hz. Combining these numerical fluctuations and the resonant phenomenon, resonance may occur under the vibration at 3.5 Hz. Thus, the natural frequency of the established lumbar FE model should be 3.5 Hz.

High axial effective stress increases the bearing burden of the annulus ground substance. Pore pressure can resist the external pressure in the intervertebral disc. If pore pressure dissipates quickly, most of the external pressure may be tolerated by the solid skeleton. Excessive radial strain in the annulus ground substance causes serious deformation of the annulus fibrosus. Under the combined influences of the above three conditions, nucleus pulposus may break through the injured annulus fibrosus, damage the intervertebral disc, and lead to lumbar degeneration in long-term effects [29, 30]. Herein, the axial effective stress, pore pressure, and radial strain peaked at 0.117 MPa, 0.322 MPa, and 14.892%, respectively, when the excitation frequency was far from the natural frequency, such as 2 Hz, 0.129 MPa, 0.237 MPa, and 16.094%, respectively, under resonant frequency. Meanwhile, the numerical fluctuation amplitudes of the lumbar mechanical parameters were great when the excitation frequency was closed to the natural frequency. Therefore, in these cases, the axial effective stress and radial strain greatly increased, and the pore pressure remarkably decreased, possibly leading to lumbar injury and degeneration. This phenomenon explained why resonant vibration seriously affects the human lumbar spine from the changes in the numerical fluctuation amplitudes.

The shapes of the resonant curves clearly differed from those of the nonresonant curves. The three lumbar mechanical parameters in the resonant curves only expressed one complete numerical changing cycle in an hour. For the excitation frequency away from the natural frequency, such as 2, 4.5, and 5 Hz, the numerical changing cycle gradually shortened as the excitation frequency increased; when the excitation frequency was close to the natural frequency, the numerical changing cycle prolonged. Meanwhile, the changes in numerical fluctuation cycle under various excitation frequencies led to different numerical fluctuation amplitudes in one cycle. Here, we considered the axial effective stress in the last cycle as an example. The stress fluctuation amplitude was 0.022 MPa at 2 Hz vibration in the last cycle, 0.024 MPa at 2.5 Hz, 0.026 MPa at 3 Hz, 0.035 MPa at 3.5 Hz, 0.028 MPa at 4 Hz, 0.020 MPa at 4.5 Hz, and 0.019 MPa at 5 Hz. The stress fluctuation amplitude in one cycle decreased as the cycle shortened. This phenomenon may be related to the continuous damping capacity and time inside the lumbar intervertebral disc. The damping effect was evident in a short cycle, directly leading to low stress fluctuation amplitude; when the vibrational cycle prolonged, the damping capacity and effect weakened due to the exuding fluid in the disc in one cycle, resulting in great stress fluctuation amplitude [4, 9]. Given that the resonant curve expressed one complete cycle in an hour, the damping effect was rather weak in the middle and late stages of loading, causing the stress to increase significantly over time in one cycle. Therefore, the occurrence of resonance may prolong the numerical changing cycle and lead to great numerical fluctuation amplitude in one cycle, ultimately changing the lumbar mechanical parameters to be detrimental to the lumbar spine health. This phenomenon explained why resonant vibration seriously affects the human lumbar spine from the changes in the numerical fluctuation cycle.

This paper has several limitations in the process of dynamic FE analysis. The boundary condition of the lumbar FE model was simplified. First, a fixed osmotic pore pressure was set at the outer surface of the spine. As the lumbar spine was compressed and the fluid in the intervertebral disc was exuded, the density of the fixed charges within the nucleus increased, causing changes in the osmotic pore pressure with interstitial fluids surrounding the disc. However, Galbusera et al. concluded that the differences between the fixed and altered osmotic pore pressure in the predicted results were minimal [31]. Second, the constraint condition for a person in sitting position under vibration was simplified, which may exert certain effects on the predicted results. The conclusions in this study were obtained by comparing the mechanical responses of different vibration frequencies. The effects of fixed osmotic pore pressure and simplified constraint condition were considered for all analyses. Therefore, the conclusions may not be significantly influenced by the simplified boundary condition. Except for the boundary condition, only one lumbar FE model was established, indicating that the predicted absolute values may not be representative for an average person. This study aimed at presenting an approach to predict natural frequency; thus, our lumbar FE model was used as an example to introduce this approach. Although the dynamic FE analysis contained assumptions, model validation ensured the accuracy of the FE model. Hence, accurate results and meaningful conclusions could be obtained from the investigation on the basis of precise modeling and analytical method.

The innovation of this approach was to predict the specific numerical value of the natural frequency of the human lumbar spine by using the porous seepage-stress coupled FE analysis [32]. Initially, a real time-dependent vibrational condition was simulated, and the changes in lumbar mechanical parameters with time were investigated. Meanwhile, the porous mechanical parameters in the lumbar spine can be predicted to analyze changes in the internal fluid properties under vibration. Accordingly, the porous seepage-stress coupled FE analysis might be applicable to predict the specific numerical value of the natural frequency of a person who has CT images with either healthy or degenerated lumbar spine.

## 5. Conclusions

This study presented an approach to predict the specific numerical value of the natural frequency of the human lumbar spine through dynamic FE analysis. Results showed that the natural frequency of the established lumbar FE model was 3.5 Hz. Meanwhile, the reasons of the resonance causing remarkable changes in the lumbar mechanical parameters were explained by comparing and analyzing the differences between the resonant and nonresonant vibrational curves and data.

## Figures and Tables

**Table 1:** The material parameters of the poroelastic lumbar FE model.

		Elastic formulation		Poroelastic formulation		References
		Elastic modulus (MPa)	Poisson's ratio	Permeability (m^4^/Ns)	Void ratio	
Cancellous bone	Linear elastic	100	0.2	1*e*^−13^	0.4	[15, 19, 23]
Cortical bone	Linear elastic	10,000	0.3	1*e*^−20^	0.02	[15, 19, 23]
Annulus fibrosus	Linear elastic	357–550	0.3			[22]
Annulus ground substance	Hyperelastic	*C*_10_ = 0.315, *D* = 0.688		9*e*^−16^	2.33	[15, 20, 24]
Nucleus pulposus	Hyperelastic	*C*_10_ = 0.125, *D* = 2.475		3*e*^−16^	4	[15, 20, 24]
Ligament/facet	Hyperelastic	Fitting from previous experiment				[21]

## Data Availability

The data used to support the findings of this study are available from the corresponding author upon request.
